# Crystal structure of *trans*-di­chlorido­(1,4,8,11-tetra­aza­undecane-κ^4^ *N*)chromium(III) perchlorate determined from synchrotron data

**DOI:** 10.1107/S2056989016002978

**Published:** 2016-02-24

**Authors:** Dohyun Moon, Jong-Ha Choi

**Affiliations:** a Pohang Accelerator Laboratory, POSTECH, Pohang 37673, Republic of Korea; b Department of Chemistry, Andong National University, Andong 36729, Republic of Korea

**Keywords:** crystal structure, 1,4,8,11-tetra­aza­undeca­ne, chloride ligand, *trans–meso* (*RS*) conformation, chromium(III) complex, hydrogen bonding, synchrotron radiation

## Abstract

The structure of the title complex, [CrCl_2_(2,3,2-tet)]ClO_4_ (2,3,2-tet is 1,4,8,11-tetra­aza­undecane, C_7_H_20_N_4_), has been determined from synchrotron data. The Cr^III^ ion is coordinated by the four N atoms of the 1,4,8,11-tetra­aza­undecane ligand in the equatorial plane and two chloride ions in an axial arrangement, displaying a slightly distorted octa­hedral coordination environment. The two H atoms of the secondary amines are grouped on the same side of the equatorial N_4_ plane (*meso*-*RS* conformation). The Cr—N bond lengths range from 2.069 (2) to 2.084 (2) Å, while the mean Cr—Cl bond length is 2.325 (2) Å. The crystal structure is stabilized by inter­molecular hydrogen-bonding inter­actions between the primary and secondary amine groups of the 2,3,2-tet ligands, the Cl ligands and the O atoms of the perchlorate counter-anion, forming corrugated layers parallel to (010).

## Abstract 2

In the title compound, [CrCl_2_(C_7_H_20_N_4_)]ClO_4_, the Cr^III^ ion is coordinated by four N atoms from the 1,4,8,11-tetra­aza­undecane ligand and two chloride ions in a *trans* geometry, displaying a distorted octa­hedral arrangement. The crystal packing is stabilized by N—HCl and N—H⋯O hydrogen bonds.

## Chemical context   

**Figure 1:**
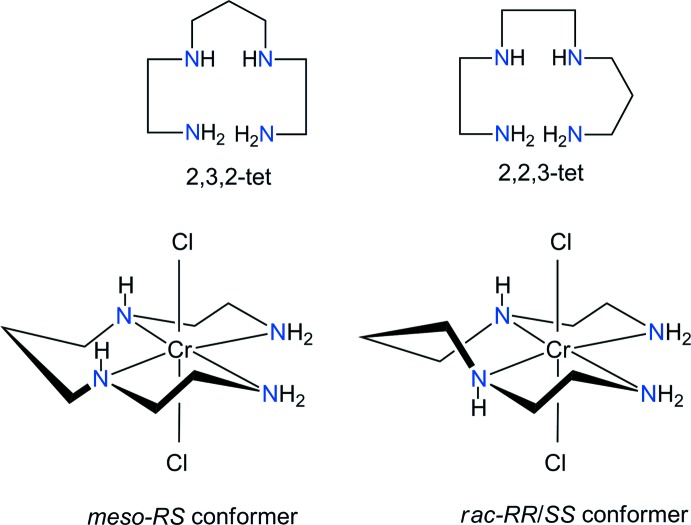
Schematic representation of the 2,3,2-tet and 2,2,3-tet ligands, and two possible conformational isomers of *trans*-[CrCl_2_(2,3,2-tet)]^+^.

Geometric and conformational isomerism in chromium(III) complexes of linear flexible tetra­dentate ligands is an inter­esting field because it has played an important role in extending the concept of stereochemistry. The 1,4,8,11-tetra­aza­undecane ligand (2,3,2-tet) is a structural isomer of 1,4,7,11-tetra­aza­undecane (2,2,3-tet). These two ligands have four nitro­gen atoms as donor groups and can adopt three different configurations in chromium(III) complexes with two additional Cl ligands (Choi *et al.*, 2008*a* ▸,*b* ▸). Two conformations of *meso-RS* or *racemic-RR/SS* isomers with respect to the orientation of the secondary amine hydrogen atoms in the *trans* isomer are also possible (Fig. 1 ▸). The two hydrogen atoms of the conformers may be on the same side (*RS*) of the equatorial N_4_ plane or on opposite sides (*RR/SS*) of this plane.
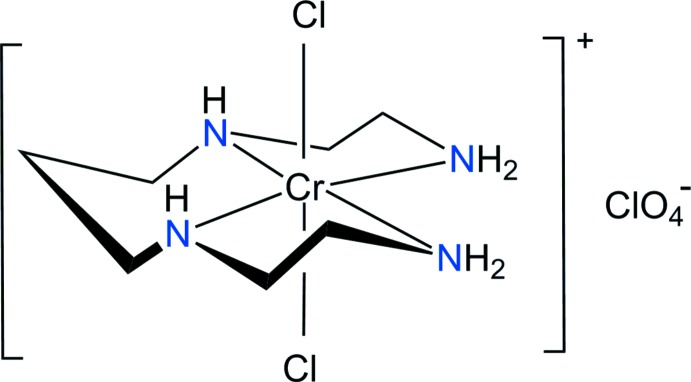


The different symmetries of transition metal complexes allow the determination of their stereochemistry from electronic absorption and infrared spectra. Indeed, infrared and electronic spectroscopic properties often are useful in determining the geometric isomers of chromium(III) complexes with linear tetra­dentate ligands (House & Garner; 1966 ▸; Kutal & Adamson, 1973 ▸; House & Yang, 1983 ▸; Kirk & Fernando, 1994 ▸). However, it should be noted that the geometric assignments based on spectroscopic studies alone are less conclusive. Both *trans* and *cis* isomers of [CrCl_2_(2,3,2-tet)]ClO_4_ have been isolated (House & Yang, 1983 ▸; Kirk & Fernando, 1994 ▸). Whereas the crystal structure and spectroscopic properties of the *cis*-*β*-di­chlorido­chromium(III) complexes containing the 2,3,2-tet ligand were reported (Choi *et al.*, 2008*b* ▸), the *trans* isomers with any anion have so far not been structurally characterized. The orientation of the secondary amine hydrogen atoms in the metal complexes is also highly relevant for medical application and likely to be a major factor in determining the anti­viral activity (Ronconi & Sadler, 2007 ▸; Ross *et al.*, 2012 ▸). In order to confirm the orientation of the secondary N—H hydrogen atoms of the Cr(III) complex with 2,3,2-tet and additional Cl ligands, we report the structure of the title compound, *trans*-[CrCl_2_(2,3,2-tet)]ClO_4_, (I), in this communication.

## Structural commentary   

**Figure 2:**
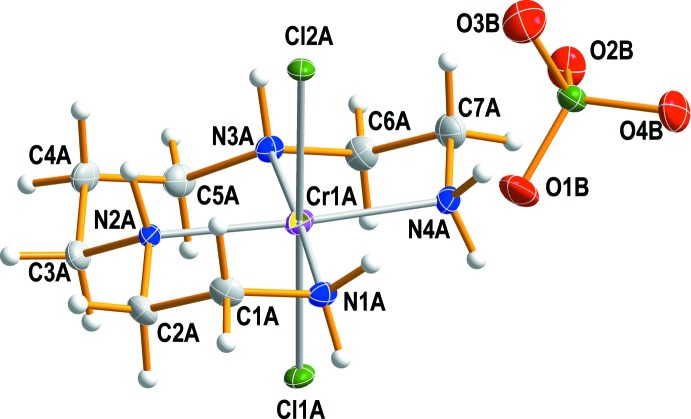
The structures of the mol­ecular components of complex (I), drawn with displacement ellipsoids at the 30% probability level.

Fig. 2 ▸ displays the mol­ecular components of compound (I). In the distorted octa­hedral complex chromium(III) cation, the four N atoms of the 2,3,2-tet ligand occupy the equatorial sites and the two chlorine atoms coordinate axially to the metal. The two hydrogen atoms of the secondary amine groups are grouped on the same side (*meso-* *RS* type) of the equatorial N_4_ plane. Such a conformation is consistent with those of *trans-*[CrF_2_(2,3,2-tet)]ClO_4_ (Bang & Pedersen, 1978 ▸) and *trans-*[Cr(NCS)_2_(2,3,2-tet)]NCS (Mäcke *et al.*, 1982 ▸). The *meso-RS* conformation may be compared with *rac-RR/SS* types of *trans*-[CrF_2_(2,2,3-tet)]ClO_4_ (Choi & Moon, 2014 ▸) and *trans*-[CrF(3,2,3-tet)(H_2_O)](ClO_4_)_2_·H_2_O (Choi & Lee, 2008 ▸).

The Cr—N bond lengths to the 2,3,2-tet ligand are in the range 2.069 (2) to 2.084 (2) Å, in good agreement with those observed in the related structures of *trans-*[CrF_2_(2,3,2-tet)]ClO_4_ (Bang & Pedersen, 1978 ▸), *trans-*[Cr(NCS)_2_(2,3,2-tet)]NCS (Mäcke *et al.*, 1982 ▸), *trans-*[CrF_2_(2,2,3-tet)]ClO_4_ (Choi & Moon, 2014 ▸), *cis*-*β*-[Cr(ox)(2,3,2-tet)]I (ox = oxalate; Kukina *et al.*, 1990 ▸) and *cis*-*β*-[Cr(N_3_)_2_(2,2,3-tet)]Br (Choi *et al.*, 2011 ▸). The two Cr—Cl distances in (I) average to 2.325 (2) Å and are close to the values found in *cis*-*β*-[CrCl_2_(2,3,2-tet)]ClO_4_ (Choi *et al.*, 2008*b* ▸) and *cis*-*β*-[CrCl_2_(2,2,3-tet)]ClO_4_ (Choi *et al.*, 2008*a* ▸). The Cr1*A*—N1*A* and Cr1*A*—N4*A* bond lengths to the primary amine N atoms are slightly longer than the Cr1*A*—N2*A* and Cr1*A*—N3*A* bond lengths to the secondary amine N atoms. It is inter­esting to note that the Cr—N bond lengths to the primary amine N atoms in *cis*-*β*-[CrCl_2_(2,3,2-tet)]ClO_4_ (Choi *et al.*, 2008*b* ▸) are slightly shorter than those to the secondary amine N atoms. Two five-membered and one six-membered chelate rings of the 2,3,2-tet ligand are present in the structure of (I). They adopt *gauche* and stable chair conformations, respectively. The bond angles of the five- and six-membered chelate rings around the chromium(III) atom are 83.72 (9) and 93.40 (9)°, respectively. The other N—C and C—C bond lengths and Cr—N—C, N—C—C and C—C—C angles are normal for a 2,3,2-tet ligand in a *gauche* or chair conformation. The tetra­hedral ClO_4_^−^ counter anion is distorted due to its involvement in hydrogen-bonding inter­actions.

## Supra­molecular features   

**Figure 3:**
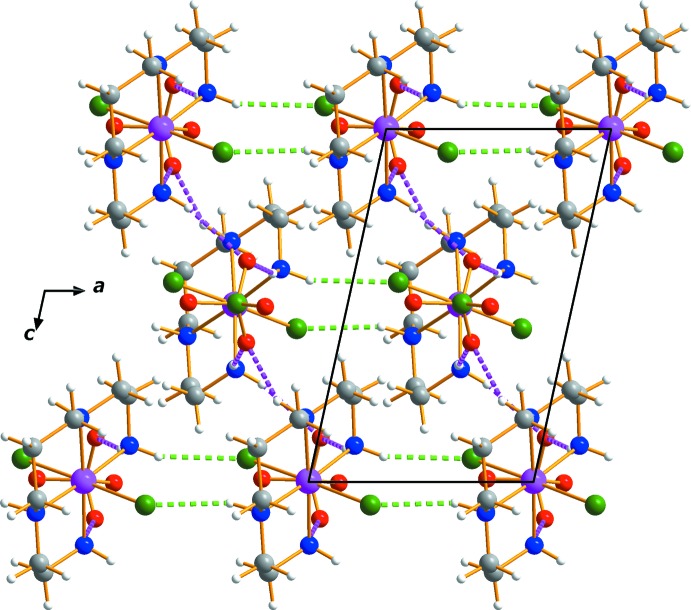
The crystal packing of complex (I) viewed perpendicular to (010). Dashed lines represent N—H⋯O (pink) and N—H⋯Cl (green) hydrogen-bonding inter­actions, respectively.

In the crystal, mol­ecules are stacked along [010]. An N—H⋯Cl hydrogen bond (N2*A*⋯Cl1*A*) links neighboring cations into rows parallel to [100] while a series of N—H⋯O contacts connect the cations to neighboring anions (Table 1 ▸). An extensive array of these contacts generates a two-dimensional network extending parallel to (010) (Figs. 3 ▸ and 4 ▸).

## Database survey   

A search in the Cambridge Structural Database (Version 5.36, last update May 2015; Groom & Allen, 2014 ▸) shows that there are four reports for Cr^III^ complexes with a [Cr*L*_2_(2,3,2-tet)]^+^ unit. The crystal structures of *trans-*[CrF_2_(2,3,2-tet)]ClO_4_ (Bang & Pedersen, 1978 ▸), *trans-*[Cr(NCS)_2_(2,3,2-tet)]NCS (Mäcke *et al.*, 1982 ▸), *cis*-*β*-[Cr(ox)(2,3,2-tet)]I (Kukina *et al.*, 1990 ▸), *cis*-*β*-[CrCl_2_(2,3,2-tet)]ClO_4_ (Choi *et al.*, 2008*b* ▸) have been reported previously. However, no structures of complexes of *trans-*[CrCl_2_(2,3,2-tet)]^+^ with any anions have been deposited.

## Synthesis and crystallization   

The free ligand 1,4,8,11-tetra­aza­undecane was purchased from Strem Chemical Company, USA. All other chemicals were reagent grade materials and were used without further purification. Compound (I) was prepared by a literature method (Kirk & Fernando, 1994 ▸). The crude perchlorate salt (0.35 g) was dissolved in 20 mL of 0.1 *M* HCl at 333 K. The filtrate was added to 5 mL of 60% HClO_4_. The resulting solution was left for slow evaporation at room temperature. Green block-like crystals suitable for X-ray structural analysis were isolated after one week. The crystals were washed with small amounts of 2-propanol and dried in air before collecting the synchrotron data.

## Refinement   

Crystal data, data collection and structure refinement details are summarized in Table 2 ▸. The H atoms were placed in geometrically idealized positions and constrained to ride on their parent atoms, with C—H distances of 0.98 Å (C—H_2_), and N—H distances of 0.90 Å and 0.99 Å (secondary amine and primary amine H atoms, respectively), with *U*_iso_(H) values of 1.2*U*_eq_ of the parent atoms.

## Supplementary Material

Crystal structure: contains datablock(s) I. DOI: 10.1107/S2056989016002978/wm5269sup1.cif

Structure factors: contains datablock(s) I. DOI: 10.1107/S2056989016002978/wm5269Isup2.hkl

CCDC reference: 1454582

Additional supporting information:  crystallographic information; 3D view; checkCIF report

## Figures and Tables

**Figure 4:**
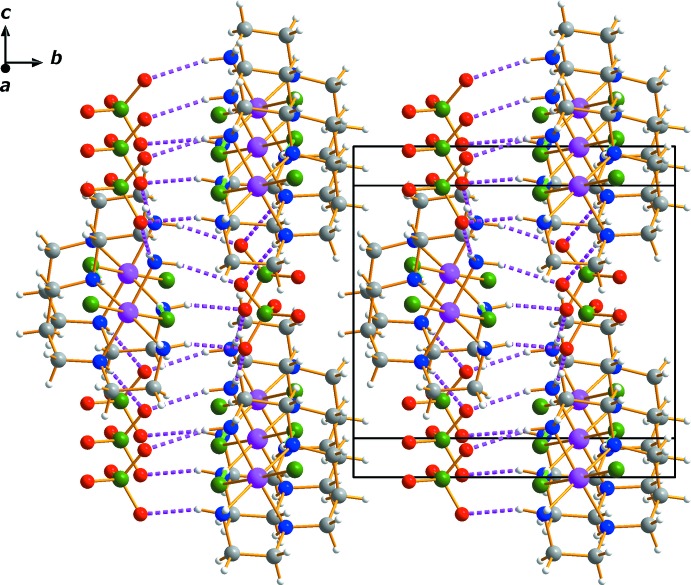
The crystal packing of complex (I) viewed approximately along [100]. The colour code is as in Fig. 3 ▸.

**Table 1:** Hydrogen-bond geometry (Å, °)

*D*—H⋯*A*	*D*—H	H⋯*A*	*D*⋯*A*	*D*—H⋯*A*
N1*A*—H1*A*1⋯O2*B* ^i^	0.90	2.30	3.187 (4)	167
N1*A*—H1*A*2⋯O1*B*	0.90	2.30	3.180 (4)	164
N2*A*—H2*A*⋯Cl1*A* ^ii^	0.99	2.47	3.332 (2)	146
N3*A*—H3*A*⋯O1*B* ^iii^	0.99	2.28	3.174 (4)	150
N4*A*—H4*A*1⋯O2*B*	0.90	2.21	3.086 (4)	163
N4*A*—H4*A*2⋯Cl2*A* ^iv^	0.90	2.56	3.405 (2)	157

Symmetry codes: (i) 
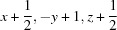
; (ii) 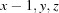; (iii) 
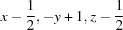
; (iv) 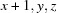.

**Table 2:** Experimental details

Crystal data	
Chemical formula	[CrCl_2_(C_7_H_20_N_4_)]ClO_4_
*M*_r_	382.62
Crystal system, space group	Monoclinic, *P* *n*
Temperature (K)	243
*a*, *b*, *c* (Å)	6.4730 (13), 11.449 (2), 10.385 (2)
β (°)	102.42 (3)
*V* (Å^3^)	751.6 (3)
*Z*	2
Radiation type	Synchrotron, λ = 0.620 Å
μ (mm^−1^)	0.89
Crystal size (mm)	0.13 × 0.13 × 0.05

Data collection	
Diffractometer	ADSC Q210 CCD area-detector
Absorption correction	Empirical (using intensity measurements) (*HKL3000sm *SCALEPACK**; Otwinowski & Minor, 1997 ▸)
*T*_min_, *T*_max_	0.893, 0.958
No. of measured, independent and observed [*I* > 2σ(*I*)] reflections	7831, 4422, 4214
*R*_int_	0.023
(sin θ/λ)_max_ (Å^−1^)	0.707

Refinement	
*R*[*F* ^2^ > 2σ(*F* ^2^)], *wR*(*F* ^2^), *S*	0.025, 0.066, 1.07
No. of reflections	4422
No. of parameters	172
No. of restraints	2
H-atom treatment	H-atom parameters constrained
Δρ_max_, Δρ_min_ (e Å^−3^)	0.39, −0.62
Absolute structure	Flack *x* determined using 2004 quotients [(*I* ^+^)−(*I* ^−^)]/[(*I* ^+^)+(*I* ^−^)] (Parsons *et al.*, 2013 ▸)
Absolute structure parameter	0.038 (9)

Computer programs: *PAL BL2D-SMDC Program* (Shin *et al.*, 2016 ▸), *HKL3000sm* (Otwinowski & Minor, 1997 ▸), *SHELXT* (Sheldrick, 2015*a* ▸), *SHELXL2014/7* (Sheldrick, 2015*b* ▸), *DIAMOND* (Putz & Brandenburg, 2014 ▸) and *publCIF* (Westrip, 2010 ▸).

## supplementary crystallographic information

### Crystal data

**Table:**

[CrCl_2_(C_7_H_20_N_4_)]ClO_4_	*F*(000) = 394
*M**_r_* = 382.62	*D*_x_ = 1.691 Mg m^−^^3^
Monoclinic, *P**n*	Synchrotron radiation, λ = 0.620 Å
*a* = 6.4730 (13) Å	Cell parameters from 22325 reflections
*b* = 11.449 (2) Å	θ = 0.4–33.6°
*c* = 10.385 (2) Å	µ = 0.89 mm^−^^1^
β = 102.42 (3)°	*T* = 243 K
*V* = 751.6 (3) Å^3^	Block, green
*Z* = 2	0.13 × 0.13 × 0.05 mm

### Data collection

**Table:**

ADSC Q210 CCD area-detector diffractometer	4214 reflections with *I* > 2σ(*I*)
Radiation source: PLSII 2D bending magnet	*R*_int_ = 0.023
ω scan	θ_max_ = 26.0°, θ_min_ = 2.3°
Absorption correction: empirical (using intensity measurements) (*HKL3000sm *SCALEPACK**; Otwinowski & Minor, 1997)	*h* = −9→9
*T*_min_ = 0.893, *T*_max_ = 0.958	*k* = −16→16
7831 measured reflections	*l* = −14→14
4422 independent reflections	

### Refinement

**Table:**

Refinement on *F*^2^	Hydrogen site location: inferred from neighbouring sites
Least-squares matrix: full	H-atom parameters constrained
*R*[*F*^2^ > 2σ(*F*^2^)] = 0.025	*w* = 1/[σ^2^(*F*_o_^2^) + (0.0435*P*)^2^] where *P* = (*F*_o_^2^ + 2*F*_c_^2^)/3
*wR*(*F*^2^) = 0.066	(Δ/σ)_max_ < 0.001
*S* = 1.07	Δρ_max_ = 0.39 e Å^−^^3^
4422 reflections	Δρ_min_ = −0.62 e Å^−^^3^
172 parameters	Absolute structure: Flack *x* determined using 2004 quotients [(*I*^+^)-(*I*^-^)]/[(*I*^+^)+(*I*^-^)] (Parsons *et al.*, 2013)
2 restraints	Absolute structure parameter: 0.038 (9)

### Special details

**Table:**

Geometry. All e.s.d.'s (except the e.s.d. in the dihedral angle between two l.s. planes) are estimated using the full covariance matrix. The cell e.s.d.'s are taken into account individually in the estimation of e.s.d.'s in distances, angles and torsion angles; correlations between e.s.d.'s in cell parameters are only used when they are defined by crystal symmetry. An approximate (isotropic) treatment of cell e.s.d.'s is used for estimating e.s.d.'s involving l.s. planes.

### Fractional atomic coordinates and isotropic or equivalent isotropic displacement parameters (Å^2^)

**Table:**

	*x*	*y*	*z*	*U*_iso_*/*U*_eq_	
Cr1A	0.49958 (5)	0.29857 (3)	0.49729 (4)	0.01583 (8)	
Cl1A	0.79710 (9)	0.18290 (6)	0.56558 (7)	0.02827 (14)	
Cl2A	0.20419 (9)	0.41841 (5)	0.43425 (6)	0.02410 (12)	
N1A	0.5655 (3)	0.3816 (2)	0.6797 (2)	0.0241 (4)	
H1A1	0.6982	0.3651	0.7229	0.029*	
H1A2	0.5535	0.4595	0.6690	0.029*	
N2A	0.3179 (3)	0.18908 (17)	0.5865 (2)	0.0199 (4)	
H2A	0.1708	0.2180	0.5598	0.024*	
N3A	0.4222 (3)	0.21127 (19)	0.3180 (2)	0.0234 (4)	
H3A	0.2837	0.2426	0.2713	0.028*	
N4A	0.6689 (3)	0.4076 (2)	0.3968 (2)	0.0249 (4)	
H4A1	0.6368	0.4828	0.4087	0.030*	
H4A2	0.8087	0.3978	0.4278	0.030*	
C1A	0.4110 (5)	0.3385 (3)	0.7556 (3)	0.0296 (6)	
H1A3	0.2754	0.3789	0.7270	0.036*	
H1A4	0.4636	0.3533	0.8499	0.036*	
C2A	0.3824 (5)	0.2085 (3)	0.7308 (3)	0.0284 (5)	
H2A1	0.5152	0.1675	0.7664	0.034*	
H2A2	0.2736	0.1783	0.7746	0.034*	
C3A	0.3107 (4)	0.0636 (2)	0.5507 (3)	0.0284 (5)	
H3A1	0.2128	0.0230	0.5955	0.034*	
H3A2	0.4515	0.0293	0.5812	0.034*	
C4A	0.2396 (5)	0.0455 (3)	0.4025 (3)	0.0335 (6)	
H4A3	0.2074	−0.0375	0.3859	0.040*	
H4A4	0.1081	0.0892	0.3713	0.040*	
C5A	0.3975 (5)	0.0824 (2)	0.3219 (3)	0.0326 (6)	
H5A1	0.5351	0.0471	0.3597	0.039*	
H5A2	0.3510	0.0531	0.2317	0.039*	
C6A	0.5783 (5)	0.2472 (3)	0.2397 (3)	0.0333 (6)	
H6A1	0.5258	0.2269	0.1467	0.040*	
H6A2	0.7126	0.2064	0.2716	0.040*	
C7A	0.6117 (5)	0.3775 (3)	0.2536 (3)	0.0352 (6)	
H7A1	0.7254	0.4016	0.2104	0.042*	
H7A2	0.4821	0.4187	0.2113	0.042*	
Cl1B	0.50722 (11)	0.72486 (5)	0.49469 (7)	0.02745 (12)	
O1B	0.5868 (6)	0.6499 (3)	0.6047 (3)	0.0654 (10)	
O2B	0.5109 (4)	0.6627 (3)	0.3746 (3)	0.0463 (6)	
O3B	0.2904 (5)	0.7539 (3)	0.4912 (3)	0.0582 (7)	
O4B	0.6361 (5)	0.8276 (2)	0.5033 (3)	0.0490 (6)	

### Atomic displacement parameters (Å^2^)

**Table:**

	*U*^11^	*U*^22^	*U*^33^	*U*^12^	*U*^13^	*U*^23^
Cr1A	0.01154 (13)	0.01462 (14)	0.01988 (14)	0.00061 (12)	0.00019 (10)	−0.00054 (13)
Cl1A	0.0156 (2)	0.0267 (3)	0.0401 (3)	0.0062 (2)	0.0006 (2)	0.0027 (2)
Cl2A	0.0175 (2)	0.0225 (3)	0.0308 (3)	0.00531 (19)	0.00184 (18)	0.0056 (2)
N1A	0.0234 (9)	0.0212 (10)	0.0244 (9)	0.0001 (8)	−0.0019 (7)	−0.0040 (8)
N2A	0.0163 (8)	0.0153 (9)	0.0270 (10)	−0.0001 (7)	0.0018 (7)	0.0032 (7)
N3A	0.0242 (10)	0.0211 (10)	0.0237 (10)	−0.0005 (8)	0.0024 (8)	−0.0044 (8)
N4A	0.0186 (9)	0.0227 (11)	0.0337 (11)	−0.0008 (7)	0.0063 (8)	0.0026 (8)
C1A	0.0364 (14)	0.0305 (15)	0.0222 (11)	0.0035 (11)	0.0071 (10)	−0.0034 (10)
C2A	0.0369 (15)	0.0236 (13)	0.0248 (12)	0.0033 (10)	0.0072 (10)	0.0056 (9)
C3A	0.0293 (12)	0.0156 (11)	0.0388 (13)	−0.0013 (9)	0.0037 (10)	0.0025 (10)
C4A	0.0333 (14)	0.0204 (12)	0.0422 (15)	−0.0079 (10)	−0.0023 (11)	−0.0055 (11)
C5A	0.0386 (15)	0.0210 (13)	0.0358 (13)	−0.0006 (10)	0.0025 (11)	−0.0097 (10)
C6A	0.0362 (15)	0.0376 (18)	0.0283 (13)	0.0012 (12)	0.0118 (11)	−0.0037 (12)
C7A	0.0369 (15)	0.0399 (18)	0.0316 (13)	−0.0016 (13)	0.0133 (11)	0.0052 (12)
Cl1B	0.0312 (3)	0.0245 (3)	0.0255 (2)	0.0024 (3)	0.00349 (19)	0.0028 (3)
O1B	0.077 (2)	0.0510 (16)	0.0495 (15)	−0.0175 (15)	−0.0279 (14)	0.0247 (13)
O2B	0.0544 (16)	0.0478 (14)	0.0396 (12)	−0.0064 (12)	0.0165 (11)	−0.0106 (10)
O3B	0.0421 (14)	0.063 (2)	0.075 (2)	0.0116 (13)	0.0243 (13)	0.0017 (16)
O4B	0.0587 (17)	0.0279 (12)	0.0574 (15)	−0.0124 (11)	0.0060 (12)	0.0005 (10)

### Geometric parameters (Å, º)

**Table:**

Cr1A—N2A	2.069 (2)	C1A—H1A4	0.9800
Cr1A—N3A	2.078 (2)	C2A—H2A1	0.9800
Cr1A—N1A	2.080 (2)	C2A—H2A2	0.9800
Cr1A—N4A	2.084 (2)	C3A—C4A	1.523 (4)
Cr1A—Cl1A	2.3191 (8)	C3A—H3A1	0.9800
Cr1A—Cl2A	2.3300 (8)	C3A—H3A2	0.9800
N1A—C1A	1.484 (4)	C4A—C5A	1.514 (4)
N1A—H1A1	0.9000	C4A—H4A3	0.9800
N1A—H1A2	0.9000	C4A—H4A4	0.9800
N2A—C3A	1.482 (3)	C5A—H5A1	0.9800
N2A—C2A	1.483 (4)	C5A—H5A2	0.9800
N2A—H2A	0.9900	C6A—C7A	1.510 (5)
N3A—C5A	1.485 (3)	C6A—H6A1	0.9800
N3A—C6A	1.485 (4)	C6A—H6A2	0.9800
N3A—H3A	0.9900	C7A—H7A1	0.9800
N4A—C7A	1.493 (4)	C7A—H7A2	0.9800
N4A—H4A1	0.9000	Cl1B—O1B	1.433 (3)
N4A—H4A2	0.9000	Cl1B—O4B	1.434 (2)
C1A—C2A	1.514 (4)	Cl1B—O3B	1.435 (3)
C1A—H1A3	0.9800	Cl1B—O2B	1.441 (3)
			
N2A—Cr1A—N3A	93.40 (9)	H1A3—C1A—H1A4	108.4
N2A—Cr1A—N1A	83.91 (9)	N2A—C2A—C1A	108.5 (2)
N3A—Cr1A—N1A	177.24 (9)	N2A—C2A—H2A1	110.0
N2A—Cr1A—N4A	176.52 (9)	C1A—C2A—H2A1	110.0
N3A—Cr1A—N4A	83.72 (9)	N2A—C2A—H2A2	110.0
N1A—Cr1A—N4A	98.95 (9)	C1A—C2A—H2A2	110.0
N2A—Cr1A—Cl1A	91.81 (6)	H2A1—C2A—H2A2	108.4
N3A—Cr1A—Cl1A	91.36 (7)	N2A—C3A—C4A	111.8 (2)
N1A—Cr1A—Cl1A	89.33 (7)	N2A—C3A—H3A1	109.3
N4A—Cr1A—Cl1A	90.21 (7)	C4A—C3A—H3A1	109.3
N2A—Cr1A—Cl2A	88.37 (6)	N2A—C3A—H3A2	109.3
N3A—Cr1A—Cl2A	90.36 (7)	C4A—C3A—H3A2	109.3
N1A—Cr1A—Cl2A	88.97 (7)	H3A1—C3A—H3A2	107.9
N4A—Cr1A—Cl2A	89.69 (7)	C5A—C4A—C3A	115.3 (2)
Cl1A—Cr1A—Cl2A	178.26 (3)	C5A—C4A—H4A3	108.5
C1A—N1A—Cr1A	107.58 (16)	C3A—C4A—H4A3	108.5
C1A—N1A—H1A1	110.2	C5A—C4A—H4A4	108.5
Cr1A—N1A—H1A1	110.2	C3A—C4A—H4A4	108.5
C1A—N1A—H1A2	110.2	H4A3—C4A—H4A4	107.5
Cr1A—N1A—H1A2	110.2	N3A—C5A—C4A	112.5 (2)
H1A1—N1A—H1A2	108.5	N3A—C5A—H5A1	109.1
C3A—N2A—C2A	112.7 (2)	C4A—C5A—H5A1	109.1
C3A—N2A—Cr1A	117.73 (18)	N3A—C5A—H5A2	109.1
C2A—N2A—Cr1A	107.34 (16)	C4A—C5A—H5A2	109.1
C3A—N2A—H2A	106.1	H5A1—C5A—H5A2	107.8
C2A—N2A—H2A	106.1	N3A—C6A—C7A	108.8 (2)
Cr1A—N2A—H2A	106.1	N3A—C6A—H6A1	109.9
C5A—N3A—C6A	112.4 (2)	C7A—C6A—H6A1	109.9
C5A—N3A—Cr1A	117.36 (17)	N3A—C6A—H6A2	109.9
C6A—N3A—Cr1A	107.22 (17)	C7A—C6A—H6A2	109.9
C5A—N3A—H3A	106.4	H6A1—C6A—H6A2	108.3
C6A—N3A—H3A	106.4	N4A—C7A—C6A	108.8 (2)
Cr1A—N3A—H3A	106.4	N4A—C7A—H7A1	109.9
C7A—N4A—Cr1A	108.43 (17)	C6A—C7A—H7A1	109.9
C7A—N4A—H4A1	110.0	N4A—C7A—H7A2	109.9
Cr1A—N4A—H4A1	110.0	C6A—C7A—H7A2	109.9
C7A—N4A—H4A2	110.0	H7A1—C7A—H7A2	108.3
Cr1A—N4A—H4A2	110.0	O1B—Cl1B—O4B	109.70 (17)
H4A1—N4A—H4A2	108.4	O1B—Cl1B—O3B	109.9 (2)
N1A—C1A—C2A	108.0 (2)	O4B—Cl1B—O3B	111.3 (2)
N1A—C1A—H1A3	110.1	O1B—Cl1B—O2B	108.91 (19)
C2A—C1A—H1A3	110.1	O4B—Cl1B—O2B	109.98 (17)
N1A—C1A—H1A4	110.1	O3B—Cl1B—O2B	106.93 (19)
C2A—C1A—H1A4	110.1		
			
Cr1A—N1A—C1A—C2A	40.4 (2)	C6A—N3A—C5A—C4A	179.6 (2)
C3A—N2A—C2A—C1A	173.1 (2)	Cr1A—N3A—C5A—C4A	54.6 (3)
Cr1A—N2A—C2A—C1A	41.9 (3)	C3A—C4A—C5A—N3A	−70.3 (3)
N1A—C1A—C2A—N2A	−55.7 (3)	C5A—N3A—C6A—C7A	−173.7 (2)
C2A—N2A—C3A—C4A	178.8 (2)	Cr1A—N3A—C6A—C7A	−43.3 (3)
Cr1A—N2A—C3A—C4A	−55.5 (3)	Cr1A—N4A—C7A—C6A	−36.0 (3)
N2A—C3A—C4A—C5A	70.5 (3)	N3A—C6A—C7A—N4A	53.5 (3)

### Hydrogen-bond geometry (Å, º)

**Table:**

*D*—H···*A*	*D*—H	H···*A*	*D*···*A*	*D*—H···*A*
N1*A*—H1*A*1···O2*B*^i^	0.90	2.30	3.187 (4)	167
N1*A*—H1*A*2···O1*B*	0.90	2.30	3.180 (4)	164
N2*A*—H2*A*···Cl1*A*^ii^	0.99	2.47	3.332 (2)	146
N3*A*—H3*A*···O1*B*^iii^	0.99	2.28	3.174 (4)	150
N4*A*—H4*A*1···O2*B*	0.90	2.21	3.086 (4)	163
N4*A*—H4*A*2···Cl2*A*^iv^	0.90	2.56	3.405 (2)	157

Symmetry codes: (i) *x*+1/2, −*y*+1, *z*+1/2; (ii) *x*−1, *y*, *z*; (iii) *x*−1/2, −*y*+1, *z*−1/2; (iv) *x*+1, *y*, *z*.
